# Comparison of maternal and fetal health outcomes in the pandemic period of covid-19 with the same last year duration in health centers of second largest city of Iran: A population-based cohort study

**DOI:** 10.1016/j.heliyon.2023.e14439

**Published:** 2023-03-10

**Authors:** Neda Davaryari, Saeed Davaryar, Adele Azarshab, Mohammad Moein Vakilzadeh, Veda Vakili, Zahra Moazzeni

**Affiliations:** a Department of Obstetrics and Gynecology, Faculty of Medicine, Mashhad University of Medical Sciences, Mashhad, Iran; b Cancer Research Center, Mashhad University of Medical Sciences, Mashhad, Iran; c Department of Community Medicine and Public Health, Mashhad University of Medical Sciences, Mashhad, Iran; d Faculty of Medicine, Mashhad University of Medical Sciences, Mashhad, Iran

**Keywords:** Pregnancy outcome, Covid-19, Pandemic

## Abstract

**Objective:**

The exact link between COVID-19 pandemic and different adverse outcomes of pregnancy remains unclear. Plus, large-scale research is lacking. In the present study, we aimed to compare the maternal and fetal health outcomes during the COVID-19 pandemic with the same last year duration in Iran.

**Design:**

Two retrospective cohorts (pre-COVID-19 and during COVID-19) were studied. The pre-COVID-19 cohort include pregnant women who had given birth between January 1, 2019 and December 31, 2019. The COVID-19 cohort, who had given birth between January 1, 2020 and December 31, 2020. The characteristics of pregnant women before COVID-19 and during COVID-19 pandemic were compared with Fisher's exact test. Univariate and multivariate log-binomial regression models were used to determine the risk ratios of the impacts of the COVID-19 pandemic on adverse pregnancy outcomes.

**Results:**

Among 128968 women showed that women who had given birth during the pandemic were more likely to be of young age, lower rates of alcohol consumption and smoking, lower weight gain, and higher rates of using synthetic milk for feeding neonates (P < 0.05). Also, the risks of preterm labor were high (cOR 95% CI, 1.13 to 1.31; p < 0.01) and the risk of caesarian were low (cOR 95% CI, 0.95 0.92 to 0.98; p < 0.01) among pregnant women who gave birth during the COVID-19 pandemic compared with those who gave birth before the pandemic.

**Conclusions:**

In summary, we found that during the COVID-19 pandemic there were the higher risks of preterm labor and lower risk of caesarean among pregnant women.

## 1 Introduction

At the end of 2019, the discovery of a new coronavirus, severe acute respiratory syndrome coronavirus-2 (SARS-CoV-2), was reported and linked to a series of pneumonia cases in Wuhan, China. The virus spread throughout the country rapidly, with several other countries following soon afterwards. The World Health Organization (WHO) named the disease Coronavirus disease 2019 (COVID-19), and declared it a global health emergency in March 2020 [1,2].

The risk of COVID-19 infection in pregnant women appears to be the same as in general population; however, it is speculated that immunological changes associated with pregnancy might leave the mother more susceptible to viral infections and their complications [3]. Indeed, some studies have demonstrated that COVID-19 infections are more severe in the final months of pregnancy [4,5]. In addition, a number of studies have indicated higher rates of adverse pregnancy outcomes in women with COVID-19, including preterm birth, cesarean section, and perinatal death [4,6,7]. COVID-19 pandemic has also seen numerous lockdown measures implemented occasionally in many countries, which could potentially disrupt maternal and neonatal health services and lead to adverse health outcomes [8].

As such, the exact link between COVID-19 pandemic and different adverse outcomes of pregnancy remain unclear, and more studies are needed to better elucidate the impacts of COVID-19 pandemic on pregnancy outcomes. Plus, large-scale research is lacking. In the present study, we aimed to compare the maternal and fetal health outcomes during the COVID-19 pandemic with the same last year duration in Iran.

## 2 Methods

### 2.1 Study settings

In Iran, maternity care is state-funded and easily accessible for everyone. Pregnant women are closely followed by health care providers in designated health centers, run under the supervision of the regional medical university, for the duration of pregnancy and a few weeks after giving birth. Their data, including past medical histories, pregnancy status and pregnancy outcomes are recorded in detail by health care staff in an electronic archive. This study was approved by the Ethics Committee of Mashhad University of Medical Sciences (IR.MUMS.REC.1399.160).

### 2.2 Study design and population

Two retrospective cohorts (pre-COVID-19 and during COVID-19) were studied. The pre-COVID-19 cohort consisted of 63618 pregnant women who had given birth between January 1, 2019 and December 31, 2019 (approximately one year before the beginning of the pandemic). There were 65350 pregnant women in the COVID-19 cohort, who had given birth between January 1, 2020 and December 31, 2020 (approximately one year after the beginning of the pandemic).

### 2.3 Data collection

Data were collected from the electronic information system of health centers of Mashhad University of Medical Sciences. These data included related demographic characteristics (age, education, and occupation), past medical & social histories, pregnancy relate data (gravidity, parity, history of previous delivery problems), and health status (body mass index (BMI) before pregnancy, gestational weight gain (GWG)). Collected maternal outcomes of pregnancy were gestational diabetes, gestational hypertension, delivery mode, hospitalization, blood transfusion, and postpartum preeclampsia. Evaluated fetal pregnancy outcomes included preterm birth, low birth weight and macrosomia.

### 2.4 Statistical analyses

The characteristics of pregnant women before COVID-19 and during COVID-19 pandemic were compared with Fisher's exact test. Univariate and multivariate log-binomial regression models were used to determine the crude risk ratios (cRRs) and adjusted risk ratios (aRRs) of the effects of the COVID-19 pandemic on poor pregnancy outcomes. Sensitivity analysis was done by fitting various models to survey the robustness of the estimation. Three models were fitted: the first model (A) was unadjusted; the second model (B) was adjusted for demographic characteristics (age, education, and occupation) and the third model (C) was based on model B, with further adjustments for pregnancy conditions (gravidity, parity, history of abortion), BMI before pregnancy, GWG, past medical and social history, history of low birth weight delivery, history of stillbirth, and history of delivery with congenital disorder. All analyses were performed using Statistical Package for the Social Sciences (SPSS) software version 26. A p value < 0.05 was considered statistically significant.

## 3 Results

A total of 128968 pregnant women were included in this study, with a mean age of 30.56 (±6.71, SD). 71.9% were unemployed and 74.4% had a bachelor's degree or less. Characteristics and pregnancy histories of the study population are provided in Table 1 and Table 2 respectively. Pregnant women who experienced the COVID-19 pandemic were more likely to be younger (30.1% compared to 25.3% in the pre-COVID-19 group), employed (28.7% compared to 25.9% in the pre-COVID-19 group), and to have received a primary school education or less (21.4% compared to 20.8% in the pre-COVID-19 group) when compared to women in the pre-pandemic group. Also, pregnant women during the COVID-19 pandemic were more likely to have a smaller number of gravidities, but a higher number of parities, compared with women in the pre-COVID-19 pandemic group. Regarding the pregnancy history, compared with women in the pre-COVID-19 pandemic group, pregnant women during the COVID-19 pandemic were more likely to have a positive history of low-birth-weight delivery (5.6% vs 6.1%, respectively), but were less likely to have a history of delivery with congenital disorder (0.9% vs 0.7%, respectively). There were not significant differences between cases in the pre-COVID-19 pandemic group and cases during the COVID-19 regarding the history of Diabetes Miletus and hypertension (all p > 0.05), but the rates of other chronic diseases were higher in COVID-19 pandemic group (5.3% vs 6.2%). Evaluating the social history of women showed that the rate of alcohol consumption and smoking were both significantly lower in pregnant women during the COVID-19 pandemic, compared with women in the pre-COVID-19 pandemic group (p < 0.01, and p = 0.002 respectively), but there were no significant differences in rates of addiction (p = 0.279). Weight gain during pregnancy was lower during the COVID-19 pandemic compared with women in the pre-COVID-19 pandemic group (63.8% vs 65.5%). Feeding neonates with synthetic milk was more common during COVID-19 pandemic, compared with pre-COVID-19 pandemic group (p = 0.001). Also, there was a significant difference between BMI index before pregnancy, There were no significant differences in other characteristics between the two groups (all p > 0.05).

**Table 1:** Characteristics of 128968 pregnant women before and during the COVID-19 pandemic.

Items		N/mean (SD)	Pre-COVID-19 (N, %; mean, SD)	COVID-19 (N, %; mean, SD)	P value
**Maternal age (years)**		30.56 (6.71)	31.09 (6.59)	30.04 (6.78)	<0.01
**Maternal age (years)**	≤24	25989	11012 (17.3)	14977 (22.9)	<0.01
	25–35	67315	33445 (52.6)	33870 (51.8)	
	≥35	35664	19161 (30.1)	16503 (25.3)	
**Occupation**	Unemployed	94,756	47521 (74.1)	47235 (71.3)	<0.01
	employed	35611	16576 (25.9)	19035 (28.7)	
**Education**	Primary school or less	27788	13481 (20.8)	14307 (21.4)	0.001
	Junior high school	29898	14692 (22.7)	15206 (22.7)	
	Senior high school	40333	20161 (31.1)	20172 (30.1)	
	Undergraduate or above	33722	16488 (25.4)	17234 (25.8)	
**Past medical history**	chronic disease	6970	3252 (5.3)	3718 (6.2)	<0.01
	Diabetes Miletus	624	286 (0.5)	56061 (0.6)	0.213^a^
	hypertension	605	288 (0.5)	318 (0.6)	0.684^a^
**Social history**	Alcohol consumption	90	75 (0.1)	15 (0.0)	<0.01^a^
	Addiction	168	89 (0.2)	79 (0.1)	0.281^a^
	Smoker	1126	601 (1.1)	525 (0.9)	0.002^a^
**Gestational weight gain**	Appropriate	62829	29721 (65.5)	33108 (63.8)	<0.01
	Inappropriate	34393	15647 (34.5)	18746 (36.2)	
**BMI before pregnancy, kg/m2**	Underweight (18.5)	7058	3312 (6.2)	3746 (6.7)	0.002
	Normal (18.5–24.9)	51968	25429 (47.6)	26539 (47.7)	
	Overweight (25–29.9)	34010	16827 (31.5)	17183 (30.9)	
	Obese (30)	16031	7847 (14.7)	8184 (14.7)	

a Fisher exact test.

**Table 2:** Pregnancy history of 128968 pregnant women before and during the COVID-19 pandemic.

Items		N/mean (SD)	Pre-COVID-19 (N, %; mean, SD)	COVID-19 (N, %; mean, SD)	P value
**Gravidity**		2.52 (1.37)	2.54 (1.36)	2.51 (1.37)	0.001
**Gravidity**	1	27403	12867 (23.6)	14536 (25.5)	<0.01
	2	36292	18043 (33.2)	18249 (32)	
	≥3	47767	23501 (43.2)	24266 (42.5)	
**parity**		2.07 (1.13)	2.05 (1.11)	2.08 (1.14)	<0.01
**Parity**	1	46097	23117 (35.7)	22980 (34.5)	<0.01
	2	47429	24182 (36.3)	23247 (35.9)	
	≥3	37749	18311 (28.3)	19438 (29.2)	
**Positive History for**	Low-birth-weight delivery	4895	2310 (5.6)	2585 (6.1)	0.001^a^
	abortion	32255	15886 (24.5)	16369 (24.5)	0.856
	Stillbirth	1250	634 (1.6)	616 (1.5)	0.459^a^
	delivery with congenital disorder	642	356 (0.9)	286 (0.7)	0.003^a^
**Gestational weight gain**	Appropriate	62829	29721 (65.5)	33108 (63.8)	<0.01
	Inappropriate	34393	15647 (34.5)	18746 (36.2)	
**Child feeding**	Mothers milk	125298	61950 (97)	63348 (96.7)	0.001
	Mothers and synthetic milk	3374	1616 (2.5)	1758 (2.7)	
	Synthetic milk	968	301 (0.5)	397 (0.6)	

a Fisher exact test.

Assessing the pregnancy outcomes demonstrated that the prevalence of caesarean sections was lower during the COVID-19 pandemic period compared with women prior to the pandemic (42.0% vs 41.3%; p = 0.007). During the COVID-19 pandemic period, the occurrence of post-partum preeclampsia was greater compared to women before the pandemic, with a difference of 0.2% (1.1% versus 0.9%), which was found to be statistically significant (p = 0.045). However, there were no significant differences in other pregnancy outcomes between the two periods. (p > 0.05, Table 3). The prevalence of preterm birth and macrosomia were higher during the COVID-19 pandemic period compared with cases prior to the pandemic (4.2% vs 4.7%, p < 0.01; and 4.3% vs 4.6%, respectively; p = 0.008). However, prevalence of low birth weight was not significantly different during the COVID-19 pandemic compared with the pre-pandemic period (p = 0.868).

**Table 3:** Pregnancy outcomes before and during the COVID-19 pandemic.

outcomes	Pre-COVID-19 (N, %; mean, SD)	COVID-19 (N, %; mean, SD)	P value
Adverse maternal outcomes			
**Gestational diabetes**	1707 (3.8)	1911 (4.0)	0.180^a^
**Gestational hypertension**	607 (0.9)	594 (0.9)	0.354^a^
Caesarean section*	25771 (42)	24649 (41.3)	0.007
**hospitalization**	5893 (9.1)	5963 (8.9)	0.256^a^
**Blood transfusion**	77 (0.1)	89 (0.1)	0.485^a^
**Postpartum preeclampsia**	574 (0.9)	622 (1.1)	0.045^a^
Adverse fetal outcomes			
**Preterm birth**	2695 (4.2)	3142 (4.7)	<0.01^a^
**Low birth weight**	4934 (7.6)	5077 (7.6)	0.868^a^
**Macrosomia**	2784 (4.3)	3058 (4.6)	0.015^a^

a Fisher exact test.

In our log-binomial regression models (Table 4), although risk for Caesarean section in univariate model was 0.97 times (95% CI, 0.92 to 0.98; p = 0.003) during the COVID-19 pandemic compared with pre-COVID-19, but after adjusting for all confounding factors, the risk was not significantly difference (p = 0.467). The risk of macrosomia during the COVID-19 pandemic compared with pre-COVID-19 cases was increased by 1.08 times (95% CI, 1.01 to 1.16; p = 0.023) when adjusting for age, occupation and education (Table 3). The risk of premature birth was increased by 1.2 times in all three models (95% CI, 1.10 to 1.31; p < 0.01).

**Table 4:** The influence of the COVID-19 pandemic on pregnancy outcomes.

Outcomes	Model A		Model B		Model C	
	cOR (95% CI)	P value	aOR (95% CI)	P value	aOR (95% CI)	P value
Adverse maternal outcomes						
**Gestational diabetes**	0.97 (0.89–1.05)	0.475	1.04 (0.95–1.12)	0.344	1.02 (0.94–1.11)	0.545
**Gestational hypertension**	0.88 (0.75–1.02)	0.109	0.94 (0.81–1.10)	0.503	0.88 (0.75–1.03)	0.115
**Caesarean section**	0.95 (0.92–0.98)	0.003	0.99 (0.96–1.02)	0.903	0.98 (0.95–1.02)	0.467
**hospitalization**	1.00 (0.95–1.05)	0.900	0.98 (0.93–1.04)	0.608	0.97 (0.92–1.03)	0.393
**Blood transfusion**	1.41 (0.91–2.19)	0.121	1.43 (0.92–2.23)	0.106	1.41 (0.90–2.19)	0.129
**Postpartum preeclampsia**	0.96 (0.82–1.12)	0.631	0.99 (0.84–1.16)	0.926	0.99 (0.84–1.16)	0.908
Adverse fetal outcomes						
**Preterm birth**	1.13 (1.13–1.31)	<0.01	1.15 (1.15–1.33)	<0.01	1.20 (1.10–1.31)	<0.01
**Low birth weight**	1.02 (0.96–1.08)	0.368	1.04 (0.98–1.10)	0.193	1.03 (0.97–1.09)	0.193
**Macrosomia**	1.07 (0.99–1.14)	0.057	1.08 (1.01–1.16)	0.023	1.06 (0.99–1.14)	0.088

Model A: a univariate model without controlling for any confounding factors.

Model B: controls for demographic characteristics (age, occupation and education).

Model C: based on model B, supplemented to control for gravidity, parity, history of abortion, BMI before pregnancy, gestational weight gain, history of chronic disease, History of Low-birth-weight delivery, History of Stillbirth, History of delivery with congenital disorder, history of Diabetes Miletus, history of hypertension, Alcohol consumption, Addiction and smoking).

aOR, adjusted odds ratio; BMI, body mass index; cOR, crude odds ratio.

## 4 Discussion

### 4.1 Summary of the findings

Results of this cohort study on 128968 women with a focus on secondary impacts of the COVID-19 pandemic on pregnancy outcomes showed that women who had given birth during the pandemic were more likely to be of young age, employed, less educated, have a smaller number of gravidity and higher number of parity, have a positive history of Low-birth-weight, less likely to have a history of delivery with congenital disorder, have higher rates of other chronic diseases, lower rates of alcohol consumption and smoking, lower weight gain, and higher rates of using synthetic milk for feeding neonates. There were the higher risks of preterm labor (13% up to 15%) and lower risk of caesarian (5%), among pregnant women who gave birth during the COVID-19 pandemic compared with those who gave birth before the pandemic.

### 4.2 Strengths and limitations

The strengths of this study include its cohort-study design, large sample size, and use of a variety variable to detect the impacts of the COVID-19 pandemic on pregnancy outcomes. Moreover, log-binomial regression models were utilized to assess the effect of either a policy intervention or a natural alteration. This multicenter cohort study was carried out in a major city of Iran. Nevertheless, there were certain limitations to our study. Firstly, it was a retrospective investigation. Moreover, we lacked access to data beyond the scope of pregnancy outcomes.

### 4.3 Comparison with other studies

Although there are conflicting reports of pregnancy outcomes during the COVID-19, but to our knowledge, this is the initial multicenter cohort study in Iran that concentrates on the secondary consequences of the COVID-19 pandemic on pregnancy outcomes. Some studies have demonstrated higher rates of gestational diabetes, hypertension, premature rupture of membrane, and admission to intensive care unit during the pandemic, compared with pre-COVID-19 period [[9], [10], [11], [12]]; while other studies have not demonstrated any such changes. A systematic review and meta-analysis by Chmielewska et al. did not find any significant increase in the prevalence of gestational diabetes, and hypertensive disorders of pregnancy during COVID-19 pandemic [13]. Yang et al. conducted a study where they reported a decrease in the unadjusted likelihood of preterm birth during the COVID-19 pandemic period in comparison to the pre-pandemic period [14]. A recent study by Du et al. found that there was a heightened chance of premature rupture of membranes and fetal distress during the COVID-19 pandemic. However, no significant connections were detected between the pandemic and other pregnancy outcomes [15].

One of the conflicting issues is the impact of COVID-19 pandemic on rates of preterm birth (PTB). In a systematic review and meta-analysis conducted by Yang et al., which analyzed the impact of COVID-19 on pregnancy and neonatal outcomes across 43 studies comprising of 986,466 women during the pandemic period and 8,716,000 women in the pre-pandemic period, it was found that the unadjusted odds of preterm birth (PTB) were lower during the pandemic period as compared to the pre-pandemic period (pooled unadjusted odds ratio [OR] 0.95, 95% confidence interval [CI] 0.93–0.98). However, when adjusted estimates were taken into consideration, which were reported by five of the studies with different factors adjusted, the pooled analysis did not reveal any significant differences in the odds of PTB during the pandemic period [14]. In the present study unadjusted odds ratio of preterm birth during COVID-19 pandemic was 1.13 (95% CI 1.13–1.31); also, the risk of premature birth was increased significantly even in adjusted models (95% CI, 1.10 to 1.31; p < 0.01). Also, the evaluation of the Yang review in birthweight of studies, shows that There was no difference in the odds of low birth weight, very low birthweight, or extremely low birth weight during COVID-19 pandemic [14]. In our study, we evaluate birth weight in two categories including macrosomia and low birth weight. Significant odds ratio was noted for macrosomia when adjusted by mother's age, occupation, and education. But other crude or adjusted models of odds ratio for low birth weight and macrosomia were not different between pre COVID-19 period compare with during COVID-19 period.

In retrospective study on 2503 pregnant women in a hospital in Tehran, Iran; Ranjbar et al. showed that the rate of preterm birth and low births weight had decreased during COVID-19 pandemic period [16]. Although their study was performed just in single center and did not evaluate the odds ratio of the pregnancy outcomes, but it was the only similar study that had been performed in the same region as of the current study.

In Du M et al. study, the prevalence of caesarean sections among pregnant women during the COVID-19-pandemic was increased. Also, they found that there was a greater proportion of women aged ≥35 years in the COVID-19 cohort [15]. In the present study, the crude odds ratio of caesarean sections had decreased only in unadjusted model had significant (cOR 0.95, 95% CI 0.92–0.98; p = 0.003) particularly those in the early stages of pregnancy, were studied during the COVID-19 pandemic period. This might be related to the recent incentive policies of childbearing in Iran.

Ceulemans et al. study on impact of the COVID-19 pandemic on breastfeeding, shows that COVID-19 pandemic has not affected breastfeeding practices, nor breastfeeding cessation [17]. In our study, feeding neonates with synthetic milk was more common during COVID-19 pandemic compared with pre-COVID-19 pandemic group.

Lebel and her collogues study on prevalence of Alcohol and substance use in pregnancy during the COVID-19 pandemic demonstrated that alcohol and substance use among pregnant Women during the pandemic was lower or comparable with overall rates of previous estimates in other samples [18]. Although in our study the number of pregnant women who drank alcohol or smoked were low both before and during COVID-19 pandemic based on self-reports, but the rates of alcohol drinking and smoking were significantly lower during pandemic (p < 0.01, and p = 0.002 respectively). Also, in our study there were no significant differences in addiction before and during the COVID-19 pandemic (p = 0.281).

In a systematic review and meta-analysis of Chmielewska and coauthors on 40 studies, the results showed no significant effects of COVID-19 pandemic on prevalence of maternal gestational diabetes, and hypertensive disorders of pregnancy [13]. In the present study, crude and adjusted odds ratio of both gestational diabetes and gestational hypertension did not significantly differ during COVID-19 pandemic compared with pre COVID-19 pandemic (p > 0.05).

The pandemic process negatively affected the daily routine and lead to sedentary life style. The sedentary lifestyle associated with obesity and get weight. In addition to, the studies indicated that obesity independently increased the risk of fetal loss [19]. However, in our study the stillbirth rates were shown as similar between the periods of study. It could be explained by the short period duration of study which could demonstrated the associated results later.

## 5 Conclusions

In summary, we found that there were more pregnant women who had given birth during the pandemic were more likely to have lower weight gain, lower rates of alcohol consumption and smoking, and. Also, there were the higher risks of preterm labor and lower risk of caesarian among pregnant women who gave birth during the COVID-19 pandemic compared with those who gave birth before the pandemic.

## Author contribution statement

Zahra Moazzeni: Conceived and designed the experiments; Contributed reagents, materials, analysis tools or data.

Neda Davaryari: Conceived and designed the experiments; Performed the experiments; Wrote the paper.

Saeed Davaryar: Performed the experiments; Analyzed and interpreted the data; Wrote the paper.

Adele Azarshab: Performed the experiments; Wrote the paper.

Mohammad Moein Vakilzadeh: Analyzed and interpreted the data; Wrote the paper.

Veda Vakili: Contributed reagents, materials, analysis tools or data; Wrote the paper.

## Funding statement

Neda Davaryari was supported by Mashhad University of Medical Sciences, Mashhad, Iran [code: 990124].

## Data availability statement

Data will be made available on request.

## Declaration of interest's statement

The authors declare no competing interests.

## References

[1] Organization W.H. (2020).

[2] Huang C. (2020). Clinical features of patients infected with 2019 novel coronavirus in Wuhan, China. Lancet.

[3] Akhtar H. (2020). COVID-19 (SARS-CoV-2) infection in pregnancy: a systematic review. Gynecol. Obstet. Invest..

[4] Knight M. (2020). Characteristics and outcomes of pregnant women admitted to hospital with confirmed SARS-CoV-2 infection in UK: national population based cohort study. BMJ.

[5] Mullins E. (2020). Coronavirus in pregnancy and delivery: rapid review. Ultrasound Obstet. Gynecol..

[6] Chen H. (2020). Clinical characteristics and intrauterine vertical transmission potential of COVID-19 infection in nine pregnant women: a retrospective review of medical records. Lancet.

[7] Allotey J. (2020). Clinical manifestations, risk factors, and maternal and perinatal outcomes of coronavirus disease 2019 in pregnancy: living systematic review and meta-analysis. BMJ.

[8] Kc A. (2020). Effect of the COVID-19 pandemic response on intrapartum care, stillbirth, and neonatal mortality outcomes in Nepal: a prospective observational study. Lancet Global Health.

[9] Justman N. (2020). Lockdown with a price: the impact of the COVID-19 pandemic on prenatal care and perinatal outcomes in a tertiary care center. Isr. Med. Assoc. J..

[10] Gu X.X. (2020). How to prevent in-hospital COVID-19 infection and reassure women about the safety of pregnancy: experience from an obstetric center in China. J. Int. Med. Res..

[11] Kugelman N. (2020). Changes in the obstetrical emergency department profile during the COVID-19 pandemic. J. Matern. Fetal Neonatal Med..

[12] Goyal M. (2021). The effect of the COVID-19 pandemic on maternal health due to delay in seeking health care: experience from a tertiary center. Int. J. Gynaecol. Obstet..

[13] Chmielewska B. (2021). Effects of the COVID-19 pandemic on maternal and perinatal outcomes: a systematic review and meta-analysis. Lancet Global Health.

[14] Yang J. (2022). COVID‐19 pandemic and population‐level pregnancy and neonatal outcomes in general population: a living systematic review and meta‐analysis (Update# 2: november 20, 2021). Acta Obstet. Gynecol. Scand..

[15] Du M. (2021). Association between the COVID-19 pandemic and the risk for adverse pregnancy outcomes: a cohort study. BMJ Open.

[16] Ranjbar F. (2021). Changes in pregnancy outcomes during the COVID-19 lockdown in Iran. BMC Pregnancy Childbirth.

[17] Ceulemans M. (2020). SARS-CoV-2 infections and impact of the COVID-19 pandemic in pregnancy and breastfeeding: results from an observational study in primary care in Belgium. Int. J. Environ. Res. Publ. Health.

[18] Kar P. (2021). Alcohol and substance use in pregnancy during the COVID-19 pandemic. Drug Alcohol Depend..

[19] Woolner A.M., Bhattacharya S. (2015). Obesity and stillbirth. Best Pract. Res. Clin. Obstet. Gynaecol..

